# Treatment of Scarlet Fever

**Published:** 1877-03

**Authors:** 


### TREATMENT OF SCARLET FEVER.

[The following communications, sent in response to requests made by the editor of the JOURNAL AND EXAMINER, are contributed by physicians engaged in large practice. Communications of this kind give the best exposition of the various methods of reaching the same object by different lines of practice.—ED.]

B. M. Griffith, M.D., Springfield, Illinois, writes: Without entering into a discussion of the etiology, history or pathological elements of scarlet fever, further than to recognize it as a virulent type of an acute, infectious disease, I will proceed at once to the management and treatment of this malady.

Fortunately there are many grades of this disease. There are many cases of a mild type which will recover without any medication, and many that require little more than the observance of hygienic laws. Again there are many cases that require the vigilance and skill of the most astute practitioner to safely conduct the victim through an attack of this disease. To that intense form of the scarlatina, I wish to offer some suggestions in the treatment which has been most successful with me.

Any practitioner of experience in this malady recognizes the fact that "there is no specific for scarlatina." Any careful observer at the bedside will also recognize the fact that those remedies most potent in treatment are those which destroy the specific poison, control the temperature and prevent the disintegration of tissue. Of such remedies, sulpho-carbolate of soda, carbolic acid, salicylic acid, iodine, bromine and chlorine are the principal ones.

Take, as a typical case, a child ten or twelve years old, of good, vigorous constitution, in which the palate, uvula and tonsils are red and swollen, with enlarged and inflamed cervical, inguinal and axillary glands, eruption well developed, high grade of fever, tongue dry, and pulse rapid. The bowels should be kept open, and, for obvious reasons, saline cathartics are preferable, like effervescing crab orchard salts or seidlitz powders. For the throat I generally give this prescription:

℞ Potass. Chlorat.............................ʒ ij.
Tinct. Ferri Mur............................℥ ss.
Syrup Simpl...............................℥ iij.
Aquæ Destil................................℥ ij.
M. S. Teaspoonful in water, and gargle once in two hours.

In the beginning cold applications to swollen glands and frequent allowance of ice, to relieve thirst and check inflammation about palate, uvula and tonsils.

℞ Acid. Salicylic. Sol........................ ℥ ij.

Potass. Chlorat............................ ʒ ij.

Aquæ Destil............................ ℥ ij.

M. S. Teaspoonful in water once in two hours.

(N. B.—The salicylic acid solution has two gr. to the ʒ, prepared with boracic acid.)

If the temperature is very high, and the thermometer registers 102° or over, give salicylic acid solution, two teaspoonfuls in water, alternating with potass. mixture until the temperature is reduced.

Salicylic acid is unquestionably antiseptic, antizymotic and antipyretic, and cannot be too highly praised as a remedy in scarlet fever, diphtheria and typhoid fever. Scarlatina must have a remedy that meets it on the very threshold of its invasion, and contends the ground step by step until conquered and utterly routed. Local remedies are only palliative and adjuvants in the treatment, and he who recognizes the constitutional malaise has already taken the initiatory step which leads to victory. Salicylic acid as a remedy meets the indications. Only such remedies as antagonize the disease element ultimately win the battle. Cases treated with this remedy have been unusually exempt from the troublesome and annoying sequelæ of this disease. Albuminuria, dropsy, purulent discharges from mucous membranes, seldom give any trouble.

As a tonic—

℞ Quiniæ Sulph............................ ʒ ss.

Tinct. Ferri Mur.......................... ʒ ij.

Potass. Chlorat............................ ʒ j.

Syrup Simpl............................ ℥ ij.

Aquæ Destil............................ ℥ ij.

M. S. Teaspoonful in water once in three hours.

Hygienic laws must be strictly enforced, not only for the benefit of the household, but for the assistance in the cure of the patient. Cleanliness, frequent change of clothing for the bed and patient, ventilation, and above all *disinfection.* For the latter vaporized iodine is altogether the best and most pleasant method—less offensive, and more tolerant by the nasal passages. Half grain of iodine, five grains of iodide of potassium, with an ounce of water, kept in an open vessel at a high temperature, will be sufficient, locally, to relieve the excessive irritation of skin.

℞ Aquæ Cologniensis.... .................. ℥ iiij.

Glycerinæ............................ ℥ ij.

M. S. Apply freely with sponge as often as required.

There are cases which from the beginning show profound depression from the poisoned condition of system.

℞ Ammon. Carb......................... ʒ iij.
Syrup. Aurant.......................... ℥ j.
Emuls. Amar. Amygd..................... ℥ iij.
M. S. Teaspoonful once in two hours, or oftener if necessary, until good circulation is restored, alternating with sol. salicylic acid, teaspoonful doses.

The salicylic acid lessens the temperature; it does so as an antiseptic and antizymotic, antagonizing the poison which is producing depression.

In those cases of painful ulceration of fauces and tonsils—

℞ Chloral. Hydrat........................ ʒ j.
Glycerinæ ............................ ʒ v.
M. S. Applied with brush, gives great comfort, enabling the patient to swallow medicine and food without so much pain.

Sol. of mur. ammon. may be substituted for the ice or cold water to glandular inflammations, by cloths dipped in the solution and applied.

F. B. Haller, M.D., of Vandalia, Illinois, says: My treatment in the main is expectant. In the early stage I usually give an emetic of ipecac, and frequently follow it with a cathartic of calomel and rhubarb. I then place them on chlorate of potash and ipecac, in small doses, at short intervals, watching the progress of the disease; treating symptoms as they are manifested. If the fever is high, give veratum viride and spts. nitr. dulcis; if the throat is sore, use a gargle of chlorate of potash or Watson's chlorine mixture. I am careful to have the room well ventilated, and keep my patients as much isolated as possible.

When desquamation sets in I put them on a liberal diet, and vigilantly watch the kidneys to keep them in as near a normal condition as may be; keep an eye to the throat and ears, so as to guard against any trouble with them.

This is an outline of my general treatment in ordinary cases.

In malignant cases of a typhoid grade, where the vitality is very low, I resort to stimulants and the supporting treatment from the beginning, I think with advantage. It is astonishing to me to see how much stimulants are required in some cases to keep up the vitality until the disease has spent itself.

I have no specific or hobby on scarlet fever. I am not much of an admirer of specifics. I believe in treating symptoms in all diseases and grades.

Lucius Clark, M.D., of Rockford, Illinois, says: As to scarlet fever and its treatment, I have no specific plan. During eruption I anoint the skin with lard freely and often. If fever is high, as shown by thermometer, I use quinine in full antipyretic doses, also veratum viride carefully. If any symptoms of special congestion of kidney, I foment the lumbar region faithfully; for gargle, if fauces inflame, I prefer bisulphite soda, used same as I formerly used chlorate potash — I fancy it is a better anti-ferment. Also I direct some of the solution to be swallowed often, unless diarrhœa is present. I want mouth and fauces washed often; and not least, full and free ventilation all of the time.

Hiram Nance, M.D., of Kewanee, says: My method of treating scarlatina is very simple indeed. Believing scarlatina to be strictly a self-limited disease, and having no confidence in any remedial agent to abbreviate its course, my only object is to render my little patient as comfortable as possible until the *vis medicatrix naturæ* accomplishes the cure. In my opinion, as a general rule, there is too much medication, and I have taken the liberty to denounce the administration of specifics and vaunted remedies that have gained the reputation of curing scarlatina, and would caution all young physicians not to place much confidence in the so-called new methods for its treatment.

An article was read before the State Medical Association, at Jacksonville, at its session in 1875, by one of our members, which related unparalleled success. I don't wish to criticise in this short article, but would say that if we could all succeed so well we need not look farther for a panacea. Better trust to continued *aqua destillata*, or any other *placebo*, than administer cathartics, emetics, and such like remedies. Let us err, if we err at all by the administration of any remedy, by not giving anything that will endanger the life of our patient.

I treat my patients, whether *simple*, *anginose* or *malignant*, nearly all alike, varying the treatment very little indeed, to suit the variety of cases and types. I feel confident that medical men have placed too much confidence in medicine in this disease; and I hope this short article may lead many young men to halt before anticipating speedy relief from some newly introduced remedy or new method of treatment.

When called to a patient, I immediately have the room properly warmed and ventilated, keeping the temperature at about 68° or 70°, with the windows let down from above; permit no drafts of wind through the room, but let the contaminated air escape from above. If we have more patients than one at the same house, let each little one have a couch or bed to itself; order all the cold water they desire, or even ice water and lemonade would not be inappropriate; also order the mother or nurse to rub the child four or five times a day thoroughly with lard, and not to be afraid of putting on too much; give no cathartic, nor permit the idea of emetic to enter my mind. Should the patient's bowels remain unmoved for four or five days, I order a mild injection, or give a small dose of magnesia, or what is probably better, give cream of tartar or *pulv. jalap. comp.*

From the time I am called until the exanthematous rash is entirely gone, I give to a child from two to three years old the following prescription:

℞ Acid. Hydro Chloric........................ ʒ j.

Syrup. Simpl.............................. ℥ ij.

Pot. Chlorat.............................. ʒ iij.

Aquæ Ros.................................. ℥ ij.

M. Sig. One large teaspoonful every two hours; for a child six or eight years old, increase the dose to half a tablespoonful.

I consider this treatment a good one; and after a practice of about thirty years, I am not disposed in the least to try any other. If the patient is restless, nervous, and can't sleep, I give tinct. opii camph., or small doses of sol. morphiæ. If cerebral symptoms manifest themselves, I would give *hyd. chloral*, combined with potassic bromide. I never use any throat wash, nor probang saturated with any astringent or escharotic, believing that the muriatic acid and chlorate potash in the medicine are sufficient to cleanse the throat in the act of deglutition.

This is my treatment in brief, varying it but little in any case during the active stage.

A physician who leaves his patient for cured, and does not give strict instructions about the future nursing, is culpable in the extreme. No patient, no matter how light the disease has been, is safe for two or three weeks, for dropsy in some form is liable to make its appearance and hurry our little patient off with hydropericardium, ascites, albuminuria or some other allied symptom. So before leaving tell the mother or nurse to keep the patient in the house for ten or fourteen days; use warm flannel clothing, and sponge the surface every three or four days until the desquamation is complete.

H. Wardner, M.D., of Cairo, Illinois, writes: In my treatment of the disease my patients were isolated as completely as the circumstances of each case would allow. Free ventilation and even temperature were insisted upon, as well as cleanliness of the patient, the clothing, and all the immediate surroundings. The air of the sick room and of the house generally was disinfected by the use of bromo-chloralum, or other non-offensive disinfectants. Soiled clothing was soaked in water medicated with carbolic acid, chloride of lime or permanganate of potash for twenty-four hours or more.

The patients were sponged during the febrile stage from two to six times in twenty-four hours, with water of a temperature to be agreeable to the patient. After the appearance of the rash they were anointed night and morning with olive oil, and this was kept up until after the desquamation was completed. When the oil was not convenient, a piece of fat bacon supplied its place. This part of the treatment I considered very important, for reasons not necessary to give here. A liniment, consisting of olive oil two parts, chloroform and aq. ammon. each one part, to which laudanum or belladonna was sometimes added, was applied to the neck and over any part where there was pain and swelling. Whenever suppuration was pretty certain, the parts were poulticed and the pus evacuated as soon as practicable. I used iodine in some cases, but did not see much benefit from it.

I used a gargle consisting of saturated solution of chlorate of potash *three parts*, glycerine *two parts*, tinct. digitalis *one* part, with a little morphine when required; also a gargle of solution of carbolic acid. These were used alternately every one or two hours, as required. Ulceration of the throat was treated by the application of tinct. chloride of iron, compound tinct. of benzoin or permanganate of potash, as required.

The internal treatment during the febrile stages consisted of very small doses of digitalis, combined sometimes with the same or smaller doses of sulphate of zinc. It is given in solution $\frac{1}{48}$ of a grain of each every two hours, amounting to $\frac{1}{4}$ of a grain in the twenty-four hours. In some cases the zinc is not well borne by the stomach, and must be left out. The tinct. of gelsemin. or spts. of nitre was given with, or alternately with the above, as indicated.

Lemonade was allowed freely where the stomach was not irritable. Where irritation of the stomach existed, equal parts of sweet milk and lime water were given as a drink in small quantities, and repeated as often as the patient desired or every hour or two.

Quinine was also an important remedy, and was used according to the indications in each individual case. In young children it was made into an ointment, and rubbed over the body in place of the olive oil.

The sequelæ were treated on general principles with tonics, alteratives and local applications, *pro re nata.*

In dropsy I used iod. potash, but more especially the "skimmed milk" treatment. I will say that in albuminuria, from any cause, I have not found a more valuable remedy.

In the mildest cases but little medical treatment was employed, attention being principally directed to the surroundings and comfort of the patient.

In all cases an even temperature of the room was kept up as far as possible, and the patient forbidden to leave the house for two weeks after the subsidence of the fever, or until after the desquamation was completed.

